# Firearm laws and the network of firearm movement among US states

**DOI:** 10.1186/s12889-021-11772-y

**Published:** 2021-10-07

**Authors:** Sae Takada, Kristen R. Choi, Shaw Natsui, Altaf Saadi, Liza Buchbinder, Molly Easterlin, Frederick J. Zimmerman

**Affiliations:** 1 Division of General Internal Medicine & Health Services Research, Department of Medicine, David Geffen School of Medicine, University of California Los Angeles, Los Angeles, CA 90024 USA; 2 U.S. Department of Veterans Affairs, Health Services Research & Development, Center for the Study of Healthcare Innovation, Implementation, & Policy, Los Angeles, CA 90073 USA; 3 University of California Los Angeles School of Nursing, Los Angeles, CA 90024 USA; 4 NYC Health + Hospitals, New York, NY 10013 USA; 5 Department of Neurology, Massachusetts General Hospital, Harvard Medical School, Boston, MA 02120 USA; 6 Center for Social Medicine and Humanities, Semel Institute for Neuroscience and Human Behavior, David Geffen School of Medicine, University of California Los Angeles, Los Angeles, CA 90095 USA; 7 Department of Pediatrics, Cedars-Sinai Medical Center, Los Angeles, CA 90048 USA; 8 Division of Neonatology, Department of Pediatrics, LAC+USC Medical Center, Keck School of Medicine, University of Southern California, Los Angeles, CA 90033 USA; 9 Department of Health Policy and Management, Fielding School of Public Health University of California, Los Angeles, 90024 USA

**Keywords:** Firearm laws, State laws, Firearm trace data

## Abstract

**Background:**

The movement of firearm across state lines may decrease the effectiveness of state-level firearm laws. Yet, how state-level firearm policies affect cross-state movement have not yet been widely explored. This study aims to characterize the interstate movement of firearms and its relationship with state-level firearm policies.

**Methods:**

We analyzed the network of interstate firearm movement using Bureau of Alcohol, Tobacco, Firearms, and Explosives firearm trace data (2010–2017). We constructed the network of firearm movement between 50 states. We used zero-inflated negative binomial regression to estimate the relationship between the number of a state’s firearm laws and number of states for which it was the source of 100 or more firearms, adjusting for state characteristics. We used a similar model to examine the relationship between firearm laws and the number of states for which a given state was the destination of 100 or more firearms.

**Results:**

Over the 8-year period, states had an average of 26 (Standard Deviation [SD] 25.2) firearm laws. On average, a state was the source of 100 or more crime-related firearms for 2.2 (SD 2.7) states and was the destination of 100 or more crime-related firearms for 2.2 (SD 3.4) states. Greater number of firearm laws was associated with states being the source of 100 or more firearms to fewer states (Incidence Rate Ratio [IRR] 0.58 per SD, *p* < 0.001) and being the destination of 100 or more firearms from more states (IRR1.73 per SD, *p* < 0.001).

**Conclusions:**

Restrictive state-level firearm policies are associated with less movement of firearms to other states, but with more movement of firearms from outside states. The effectiveness of state-level firearm-restricting laws is complicated by a network of interstate firearm movement.

**Supplementary Information:**

The online version contains supplementary material available at 10.1186/s12889-021-11772-y.

## Background

Studies on firearm laws and firearm-related violence have focused on the association between the rates of firearm-related violence within the state and the aggregate number [1] or categories of state-level firearm laws [2–6]. A recent systematic review found that stronger state-level firearm laws are associated with reductions in firearm-related homicide rates; however, it also found inconclusive and conflicting results for many of the different categories of laws [7].

The extent to which states can regulate firearm-related violence with state-level firearm laws depends on their ability to regulate the firearms within their borders. However, firearms move across state borders [8, 9], and this movement may be due in part to the firearm laws themselves. For example, the implementation of a law limiting handgun purchases in Virginia resulted in a lower proportion of crime-related firearms recovered in the entire Northeast region that were traced to Virginia [10]. States with more stringent firearm laws in general have a higher proportion of crime-related firearms originating from outside the state [8, 11], of which a large proportion are from states with weaker firearm laws [12]. For pairs of states, increasing firearm law stringency in the source state was associated with reduced movement of firearms between two states, while increasing stringency in the destination state lead to increased movement [9, 13].

This study aims to build upon the literature on interstate firearm movement by describing the analysis of the network of crime-related firearm movement between states over an eight-year period. Prior studies have relied on measuring the proportions of firearms originating from source states, or the relative differences in state-level firearm laws between two states and the movement of firearms between them. However, these have a limited ability to examine the dynamic interplay of what is happening among all states at the same time. The network approach allows us to examine the relationships among all states simultaneously and study the movement of firearms both into and out of each state. We hypothesize that states with fewer firearm laws serve as source states of crime-related firearms recovered in other states, and that states with more firearm laws serve as destination states of crime-related firearms from other states.

## Methods

### Study design and data source

This is an analysis of state-level data from 2010 to 2017. The study was determined to be exempt from Institutional Review Board regulation at the University of California, Los Angeles because it uses de-identified, publicly available, state-level data.

### Network of firearm movement

We constructed the network of firearm movement between 50 states using publicly available firearm trace data (2010–2017) from the Bureau of Alcohol, Tobacco, Firearms, and Explosives (ATF) at the time this study was conducted. The ATF maintains a database of firearms used in crimes which are successfully traced to their original point of purchase [14]. The ATF conducts firearm tracing at the request of law enforcement agencies engaged in a criminal investigation in which a firearm has been used or is suspected to have been used, with the intent to link a suspect to a firearm and identify trends and pattens in the movement of illegal firearms. Using these data, we defined a network tie between two states when there was movement of 100 or more firearms in a given year from the state where the firearm is purchased (“source state”) to the state where the firearm is recovered (“destination state”). A state could serve as both a source state and a destination state for another state if it was both a source and destination for 100 or more firearms in a given year. A network of firearm movement was constructed for each year, for a total of 8 networks. For each state, we calculated the number of states for which it served as source state (outdegree) and the number of states for which it served as destination state (indegree). Sensitivity analyses were conducted by constructing the network of firearm movement using cut-offs of 50, 60, 70, 80, and 90 or more firearms.

### Dependent variables

The primary dependent variables were the number of states for which the index state was the source of 100 or more crime-related firearms in a given year (outdegree) and the number of states for which the index state was the destination of 100 or more crime-related firearms in a given year (indegree), which were calculated based on the networks described above.

### Independent variables

Following other studies [1, 11, 15], the independent variable was the firearm law strength score, an unweighted count of state-level firearm laws. This variable was obtained from the State Firearm Laws Database, which compiles data on state-level laws in all 50 states since 1991 and codes them into fourteen categories of laws that regulate and deregulate firearms [16]. Laws regulating firearms include those regulating dealers and buyers, those regulating possession of firearms, those regulating purchase or possession of assault weapons, and those preventing individuals with a history of crime, domestic violence, and mental health conditions from possessing firearms. Laws deregulating firearms include laws providing blanket immunity to firearm manufacturers and Stand-Your-Ground laws that allow individuals to use firearms with immunity from the law when they can claim a self-defined need to protect their property. A higher firearm-law strength score denotes more laws regulating firearms and fewer laws deregulating firearms. This variable was scaled to have a mean of 0 and a standard deviation of 1.

We examined all state-level firearm laws rather than limiting the focus to those that specifically prohibit firearm trafficking by regulating the purchase of firearms. Prior studies have found that numerous categories of state-level firearm laws, ranging from those that regulate purchase or registration of firearms, those that regulate concealed carry permits, to those that allow municipalities and cites to regulate firearms, were associated with less interstate movement of crime-related firearms [9, 10, 17, 18]. Further, studies interviewing persons incarcerated for firearm-related offenses showed that the majority of firearms were acquired through their friends, acquaintances, family members, and other members of their social network [19, 20]. Such transactions in the secondary firearm market were unreported and remained unregulated by laws targeting firearm trafficking, especially as many laws exempt the transfer or sale of the firearm to relatives.

### Covariates

We used the following state-level data to adjust for characteristics previously associated with firearm-related violence: poverty rate [21–23], a validated proxy measure for state-level firearm ownership [24], and county-weighted state density as a proxy for the average urbanicity of the state (the sum across all counties in the state of [county population / county land area] * [county population / state population]) [21, 23, 25]. The proxy measure of state-level firearm ownership developed by Siegel and colleagues [24] uses the proportion of firearm suicides in a state and per capita number of hunting licenses [26], and is highly correlated with survey-measured, household firearm ownership at 0.95. We also adjusted for state area and census division [23] to account for differences in firearm movement by state size and geographic location. These variables were scaled to have a mean of 0 and a standard deviation of 1.

### Data analysis

The distribution of the number of states for which the index state serves as the source or destination of 100 or more crime-related firearms is skewed to the right and contains a large proportion of zeros (Additional file 1: Figure S1). Therefore, we fitted zero-inflated negative-binomial models, which is designed to address overdispersion and excess zeros when analyzing count data. A Poisson model assumes that the conditional mean is equal to the conditional variance. Negative binomial models are modified Poisson models that relax this assumption, allowing the conditional mean and variance to be estimated separately, and is used for modeling over-dispersed count variables. Zero-inflated models assume that there are two latent groups: observations that necessarily have a high probability of a zero outcome (excess zeros) because of some underlying attributes, and observations that might have a zero, but might have a positive count with nonzero probability [27, 28]. In the context of this analysis, firearm movement, say, from Alaska to Florida is likely to be an excess zero because of the distance involved, while movement from Georgia to Florida is likely to be driven by policy. Zero-inflated models consist of two parts, a binary model and count model (in our case, negative binomial model) to account for excess zeros [28, 29]. We fitted a fixed-effects zero-inflated negative-binomial model to estimate the association between the number of firearm laws and the number of states for which the index state serves as the source of 100 or more crime-related firearms in a given year, adjusting for the covariates listed above. We subsequently fitted a zero-inflated negative-binomial model to estimate the association between the number of states for which the index state is the destination of 100 or more crime-related firearms in a given year, adjusting for covariates.

To test the sensitivity of our results against the choices for our methodologic approaches, sensitivity analyses were conducted. First, we fitted similar zero-inflated negative binomial regression models using the cut-offs of 50, 60, 70, 80, and 90 or more firearms. Second, we fitted similar negative binomial regression models without the zero inflation. Finally, in order to account for the serial autocorrelation between data, we fitted linear regression models with a Prais-Winsten estimator to model the association between the number of firearm laws and the log-transformed non-zero counts of states for which the index state serves as the source of 100 or more crime-related firearms or as the destination of 100 or more crime-related firearms, adjusting for covariates [30]. The networks were constructed and analyzed using *igraph* version 1.2.4.1 and *sna* version 2.4 packages, and linear regression with Prais-Winsten estimator was conducted using *prais* version 1.1.1 package for R. Zero-inflated negative-binomial regression with robust confidence intervals and negative binomial regression analyses were conducted using Stata version 16.

## Results

**Fig. 1:**
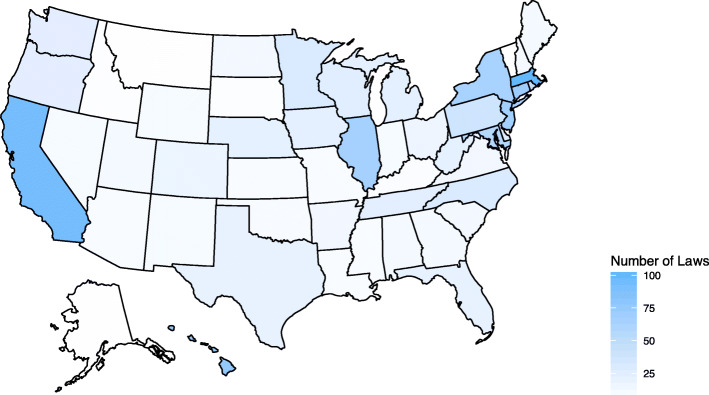
The number of state-level firearm laws in the US, 2010. We constructed the figure using *usmap* version 0.50 and *gglpot2* version 3.3.2 for R

Table 1 presents the results of descriptive characteristics. States had an average of 26 (Standard Deviation [SD] 25.2) firearm laws, ranging from two laws (Idaho, Mississippi and Missouri in 2017) to 106 laws (California in 2017) (Fig. 1). On average, a state was the source of 100 or more crime-related firearms for 2.2 (SD 2.7) states. This ranged from Texas in 2017, which was the source of 100 or more crime-related firearms to 15 states, to 142 observations over eight years (36%) in which a state was the source of 100 or more crime-related firearms to no other states that year. On average, a state was the destination of 100 or more crime-related firearms for 2.2 (SD 3.4) states. This ranged from California in 2017, which was the destination for 100 or more crime-related firearms from 22 states, to 181 observations (45%) over eight years in which a state was the destination of 100 or more crime-related firearms from no states that year.

**Table 1:** Descriptive statistics of state-level characteristics (2010–2017)

Variable		Mean (SD) or N
Poverty rate (%)		17.9 (4.3)
County-weighted density		1.08 (2.24)
Area (km2)		196,667 (249856)
Firearm ownership		16.22 (21.94)
Number of firearm laws		25.99 (25.23)
	0–9	98
	10–19	115
	20–29	77
	30–39	31
	40–49	14
	50–59	4
	60–69	23
	70 or more	38
States that serve as source for 100 or more firearms to	0 states	142
	1 state	76
	2 states	60
	3 states	34
	4 states	21
	5 or more	67
States that serve as destination for 100 or more firearms to	0 states	181
	1 state	66
	2 states	52
	3 states	17
	4 states	18
	5 or more	66

**Fig. 2:**
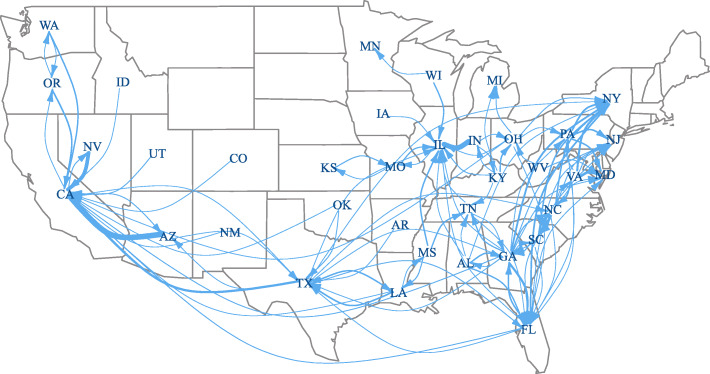
Movement of crime-related firearms across states (2010–2017), when the annual average is 100 or greater firearms. The arrow indicating movement from Arizona to California and from Indiana to Illinois are obscured due to the thickness of the line. We constructed the figure using *igraph* version 1.2.4.1 and *maps* version 3.3.0 for R

The network of interstate firearm movement is depicted in Fig. 2, which shows the average movement of firearms across states over 8 years, when the average is 100 or more firearms. The width of the arrow between two states is proportional to the average number of firearm movement between those states. States that do not have an average of 100 or more firearms move across its borders are excluded from this figure, as are New Hampshire and Massachusetts, which are connected to each other but not to other states. The highest volume of firearm movement occur between neighboring states: Arizona to California (1332 firearms); Indiana to Illinois (1173 firearms); Nevada to California (850 firearms); Virginia to Maryland (581 firearms); Georgia to Florida (499); South Carolina to North Carolina (474), Pennsylvania to New York (382 firearms) and New Jersey (356 firearms); Oregon to California (355 firearms). The exceptions are the movement from Texas to California (523 firearms), Virginia to New York (451 firearms), Georgia to New York (391 firearms), and Florida to New York (360 firearms). In addition, there is movement across long distances, going north from Georgia to New Jersey (181 firearms) and Texas to New York (106 firearms), and west from Louisiana to California (123 firearms) and Ohio to California (120 firearms). The general pattern evoked by Fig. 2 is of gun flows from low-regulation states in the south and southwest to high-regulation states.

Table 2 presents the results of the multivariable zero-inflated negative binomial analysis estimating the relationship between firearm laws in a state and the number of states for which it serves as the source of 100 or more crime-related firearms. Each additional standard deviation in the number of firearm laws was associated with 42% fewer states to which a given state is the source of 100 or more crime-related firearms (Incidence Rate Ratio [IRR] = 0.58; b = -0.55, 95% confidence interval [CI] -0.71,  -0.38, *p* < 0.001), adjusting for covariates. Each additional standard deviation in the firearm ownership was associated with 37% fewer states to which it is the source of 100 or more crime-related firearms (IRR = 0.63; b = -0.46, 95% CI -0.58, -0.35, p < 0.001).

**Table 2:** Zero-inflated negative binomial model describing the relationship between a state’s total number of firearm laws and number of states to which it is the source of 100 or more crime-related firearms in a given year, adjusting for state-level characteristics, 2010–2017

**Negative binomial model**		
**Variable**	**b (95% CI)**	**Incidence Rate Ratio**
Intercept	−1.15 (−1.64, −0.66)***	0.32
Year	0.11 (0.08, 0.14)***	1.11
Poverty rate	0.13 (0.04, 0.22)**	1.14
Density	−0.15 (−0.27, −0.03)*	0.86
Area	0.90 (0.74, 1.06)***	2.46
Firearm ownership	−0.46 (−0.58, −0.35)***	0.63
Number of laws	−0.55 (−0.71, − 0.27)***	0.58
Census division		
New England	Ref	
Mid-Atlantic	2.41 (1.77, 3.05)***	11.13
East North Central	1.88 (1.37, 2.39)***	6.54
West North Central	0.28 (−0.31, 0.88)	1.33
South Atlantic	2.39 (1.91, 2.87)***	10.92
East South Central	1.69 (1.17, 2.20)***	5.40
West South Central	0.63 (0.03, 1.23)*	1.87
Mountain	0.16 (−0.36, 0.69)	1.18
Pacific	1.83 (1.34, 2.32)***	6.26
**Zero-inflation model**		
**Variable**	**b (95% CI)**	**Adjusted Odds Ratio**
Intercept	−2.42 (−3.01, −1.83)***	0.09

* *p* < 0.05, ** *p* < 0.01, *** *p* < 0.001

Multivariable zero-inflated negative-binomial analysis estimating the relationship between firearm laws and the number of states to which a state is the destination of 100 or more crime-related firearms is shown in Table 3. Each additional standard deviation in the number of firearm laws in a state is associated with 73% more states for which it is the destination of 100 or more crime-related firearms (IRR = 1.73; b = 0.55, 95% CI 0.44, 0.65, *p* < 0.001), adjusting for covariates. Each additional standard deviation in firearm ownership was associated with 70% fewer states to which it is the destination of 100 or more crime-related firearms (IRR = 0.30; b = -1.20, 95% CI -1.48, -0.93, p < 0.001).

**Table 3:** Zero-inflated negative binomial model describing the relationship between a state’s total number of firearm laws and number of other states for which it is the destination of 100 or more crime-related firearms in a given year, adjusting for state-level characteristics, 2010–2017

**Negative binomial model**		
**Variable**	**b (95% CI)**	**Incidence Rate Ratio**
Intercept	−2.86 (−3.41, −2.31)***	0.06
Year	0.10 (0.07, 0.13)***	1.11
Poverty rate	0.22 (0.12, 0.33)**	1.25
Density	0.004 (−0.05, 0.05)	1.00
Area	0.34 (0.17, 0.50)***	1.40
Firearm ownership	−1.20 (−1.48, −0.93)***	0.30
Number of laws	0.55 (0.44, 0.65)***	1.73
Census division		
New England	Ref	
Mid-Atlantic	2.91 (2.37, 3.45)***	18.30
East North Central	3.46 (2.92, 3.99)***	31.73
West North Central	2.74 (2.07, 3.42)***	15.56
South Atlantic	3.32 (2.80, 3.83)***	27.56
East South Central	2.96 (2.33, 3.59)***	19.23
West South Central	2.59 (1.95, 3.23)***	13.38
Mountain	1.73 (1.13, 2.33)**	5.62
Pacific	2.28 (1.67, 2.90)***	9.80
**Zero-inflation model**		
**Variable**	**b (95% CI)**	**Adjusted Odds Ratio**
Intercept	−1.96 (−2.47, −1.45)***	0.14

* *p* < 0.05, ** *p* < 0.01, *** *p* < 0.001

Sensitivity analyses with multivariable zero-inflated negative-binomial models using the cut-offs of 50, 60, 70, 80, and 90 firearms showed similar results to models using the cut off of 100 (Appendix Tables 4 and 5 for cut off of 50, results of other models not shown). For analyses estimating the relationship between firearm laws and the number of states for which a state serves as the source of crime-related firearms, the coefficient on firearm laws lost significance when a cut-off of 50 or 60 firearms is used. Cut-off values of 70 and above produced statistically significant results as in the main analysis. For analyses estimating the relationship between firearm laws and the number of states for which a state is the destination of crime-related firearms, the association remained robust across all cut offs. Sensitivity analyses fitting negative binomial regression models without zero inflation (Appendix Tables 6 and 7), and sensitivity analyses fitting linear regression models with a Prais-Winsten estimator to log-transformed non-zero counts of states showed similar results to the zero-inflated negative binomial models (Appendix Tables 8 and 9).

## Discussion

Using longitudinal data on crime-related firearm tracing and state-level firearm laws, we constructed a network of firearm movement across US states from 2010 to 2017, demonstrating associations between state-level firearm policy and movement of firearms into and out of states. Consistent with our hypothesis, more firearm laws in a state was associated with both it being the source of crime-related firearms to fewer states, and the destination of crime-related firearms from more states. The estimated associations were statistically significant, and robust to the inclusion of multiple covariates including state-level poverty, density, and a firearm ownership proxy.

Our findings corroborate earlier studies showing that a passage of a single law in one state can impact the movement of firearms into and out of the state. After the implementation of a one handgun per month law in Virginia in 1993, the crime-related firearms recovered in a Northeast state was less likely to be traced to Virginia compared to other Southeast states [10]. Similarly, after the passage of Brady Bill, which instituted mandatory background checks, there was a large reduction in firearms recovered in Chicago originating from states that were not conducting background checks prior to the Brady Bill [31]. Studies among incarcerated people have found that crime-related firearms are often obtained in illegal secondary markets composed of social network members [19, 20]. Our study suggests that increasing firearm laws may decrease firearms that are available for transfer in the illegal firearm market.

Further, we found that more firearm laws was associated with a state being the destination of 100 or more crime-related firearms from more states. This is consistent with studies examining pairs of states that showed the differential in firearm laws between source and destination states correlated with the movement of firearms between the two states, such that firearms are more likely to move from states with weaker laws to states with stricter laws [9, 13]. Similarly, a study of 25 US cities found that cities in states with mandatory registration and licensing systems had a higher proportion of crime-related firearms originating from other states [8], and that states with higher number of firearm laws had a higher percentage of crime-related firearms originating from other states [11]. Our findings may lead to the question of whether the protective effect of strict firearm laws on fiream violence prevention is negated by interstate firearm movement. Although we are unable to answer this question comprehensively, data on firearm-related homicide in the US states suggest that this is not the case. From 2010 to 2017, the states identified in our analysis as sources of 100 or more crime-related firearms had an average, age-adjusted, firearm-related homicide rate of 4.21 (SD 2.20) per 100,000 population, while states that were destinations of 100 or more crime-related firearms had an average, age-adjusted, firearm-related homicide rate of 4.50 (SD 2.15) per 100,000 population [32]. The above data provide a signal that firearm laws have a protective effect on firearm-related violence despite interstate firearm movement, and that laws that reduce interstate firearm movement would reduce firearm-related violence even further. These relationships should be explored in greater depth in future research.

We also found that the firearm ownership in a given state was associated with both having fewer states for which it is a source of firearms and having higher odds of not being a destination of firearms from other states, after adjusting for the number of firearm laws and covariates. Recent studies have begun to examine the independent effects of state-level firearm laws and firearm ownership on firearm-related outcomes. A study showed an additive relationship between them, in which state-level firearm permissiveness and firearm ownership were both associated with a higher rate of mass shootings [33], while another study showed a moderating relationship in which state-level firearm policy strength was inversely associated with suicide rates in states with higher levels of firearm ownership [34].

## Conclusion

### Strengths and limitations

Interpretation of our findings are subject to limitations. This is an associative study, and as such we are unable to establish causality between firearm laws and movement of crime-related firearms. Our gun ownership variable was a proxy measure and we did not have detailed data on actual gun ownership at the state level. As an ecological analysis, the study cannot make any causal claims at the individual level, but instead points to policy factors associated with state-level firearm movement. The firearm trace data used in our analysis only included firearms used in crimes that were recovered by law enforcement, not all firearms or all firearms used in crimes. Finally, this study did not assess the impact of interstate firearm movement on negating the effects of stricter firearm laws, nor how such a relationship could affect overall state rates of firearm-related violence. Strengths of the study include using longitudinal data over an eight-year period and including all 50 states to give a more complete picture of how firearms are shared among states.

Taken together, these results suggest that the effectiveness of firearm-restricting policies at the state level is complicated by a network of firearm movement among states. These results suggest that firearms travel in complicated webs among states, and therefore, state-level firearm policies may not sufficiently address firearm availability within states. This may in part explain why certain firearm laws have not been found to have the intended effects on firearm-related violence: a recent systematic review showed that state-level laws that aim to curb firearm trafficking through regulating firearm dealers and mandating theft reporting showed conflicting and inconclusive results on the state’s firearm-related violence [7]. Our study suggests the need for federal or regional firearm laws that may better regulate crime-related firearms that move across states.

### Supplementary Information

**Additional file 1: Figure S1.** Histograms depicting the distribution of (**A**) the number of states for which states serve as sources of 100 or more firearms, (**B**) the number of states for which states serve as destinations of 100 or more firearms, (**C**) the number of states for which states serve as sources of 50 or more firearms, and (**D**) the number of states for which states serve as destinations of 50 or more firearms.

## Data Availability

The datasets analyzed during the current study are available in the State Firearm Laws (http://www.statefirearmlaws.org/) [16], Alcohol, Tobacco, Firearms and Explosives (https://www.atf.gov/resource-center/firearms-trace-data-2017) [14], U.S. Census (https://www.census.gov/) [23], and U.S. Fish & Wildlife Service (https://www.fws.gov/wsfrprograms/Subpages/LicenseInfo/Hunting.htm) [26].

## Appendix

**Table 4:** Zero-inflated negative binomial model describing the relationship between a state’s total number of firearm laws and number of other states for which it is the source of 50 or more crime-related firearms in a given year, adjusting for state-level characteristics, 2010–2017

**Negative binomial model**		
**Variable**	**b (95% CI)**	**Incidence Rate Ratio**
Intercept	−0.62 (−0.96, −0.28)***	0.54
Year	0.09 (0.06, 0.13)***	1.10
Poverty rate	0.33 (0.20, 0.46)***	1.39
Density	−0.08 (−0.19, 0.04)	0.92
Area	0.21 (0.08, 0.35)**	1.24
Firearm ownership	−0.35 (−0.46, −0.25)***	0.70
Number of laws	−0.07 (−0.21, 0.08)	0.94
Census division		
New England	Ref	
Mid-Atlantic	1.96 (1.21, 2.71)***	7.08
East North Central	2.19 (1.84, 2.53)***	8.93
West North Central	0.90 (0.47,1.33)***	2.46
South Atlantic	2.28 (1.89, 2.66)***	9.74
East South Central	2.03 (1.62, 2.43)***	7.59
West South Central	1.60 (1.15, 2.05)***	4.97
Mountain	0.95 (0.56, 1.34)***	2.59
Pacific	1.19 (0.77, 1.61)***	3.29
**Zero-inflation model**		
**Variable**	**b (95% CI)**	**Adjusted Odds Ratio**
Intercept	−10.05 (−1027.36, 1007.26)	4.32 × 10^−05^

* *p* < 0.05, ** *p* < 0.01, *** *p* < 0.001

**Table 5:** Zero-inflated negative binomial model describing the relationship between a state’s total number of firearm laws and number of other states for which it is the destination of 50 or more crime-related firearms in a given year, adjusting for state-level characteristics, 2010–2017

**Negative binomial model**		
**Variable**	**b (95% CI)**	**Incidence Rate Ratio**
Intercept	−0.80 (−1.37, − 0.22)**	0.45
Year	0.06 (0.03, 0.08)***	1.06
Poverty rate	0.11 (−0.01, 0.22)	1.11
Density	−0.03 (− 0.07, 0.01)	0.97
Area	0.99 (0.68, 1.29)***	2.68
Firearm ownership	−0.72 (− 0.86, − 0.59)***	0.48
Number of laws	0.38 (0.31, 0.45)***	1.46
Census division		
New England	Ref	
Mid-Atlantic	2.53 (1.96, 3.11)***	12.59
East North Central	2.63 (2.09, 3.17)***	13.92
West North Central	1.30 (0.65, 1.94)***	3.65
South Atlantic	2.67 (2.14, 3.19)***	14.40
East South Central	2.21 (1.64, 2.79)***	9.14
West South Central	1.00 (0.07, 1.93)*	2.71
Mountain	0.83 (0.20, 1.47)*	2.30
Pacific	1.33 (0.66, 2.00)***	3.79
**Zero-inflation model**		
**Variable**	**b (95% CI)**	**Adjusted Odds Ratio**
Intercept	−2.46 (−3.03, −1.90)***	0.09

* *p* < 0.05, ** *p* < 0.01, *** *p* < 0.001

**Table 6:** Negative binomial model describing the relationship between a state’s total number of firearm laws and number of other states for which it is the source of 100 or more crime-related firearms in a given year, adjusting for state-level characteristics, 2010–2017

Negative binomial model		
**Variable**	**b (95% CI)**	**Incidence Rate Ratio**
Intercept	−1.69 (−2.32, −1.07)***	0.18
Year	0.12 (0.08, 0.16)***	1.13
Poverty rate	0.25 (0.14, 0.37)***	1.29
Density	−0.11 (−0.25, 0.03)	0.89
Area	0.09 (−0.01, 0.20)	1.10
Firearm ownership	−0.57 (−0.74, −0.40)***	0.57
Number of laws	−0.40 (−0.55, −0.25)***	0.67
Census division		
New England	ref	
Mid-Atlantic	2.23 (1.47, 2.98)***	9.26
East North Central	2.14 (1.48, 2.79)***	8.47
West North Central	0.88 (0.16,1.59)*	2.40
South Atlantic	2.47 (1.84, 3.11)***	11.87
East South Central	1.85 (1.17, 2.54)***	6.37
West South Central	1.65 (0.95, 2.34)***	5.18
Mountain	0.97 (0.28, 1.65)**	2.63
Pacific	1.99 (1.28, 2.71)***	7.34

* *p* < 0.05, ** *p* < 0.01, *** *p* < 0.001

**Table 7:** Negative binomial model describing the relationship between a state’s total number of firearm laws and number of other states for which it is the destination of 100 or more crime-related firearms in a given year, adjusting for state-level characteristics, 2010–2017

Negative binomial model		
**Variable**	**b (95% CI)**	**Incidence Rate Ratio**
Intercept	−3.07 (−3.73, −2.41)***	0.05
Year	0.13 (0.09, 0.16)***	1.13
Poverty rate	0.35 (0.24, 0.47)***	1.42
Density	−0.02 (−0.09, 0.05)	0.98
Area	0.51 (0.39, 0.64)***	1.67
Firearm ownership	−1.23 (−1.50, −0.96)***	0.29
Number of laws	0.65 (0.52, 0.78)***	1.92
Census division		
New England	ref	
Mid-Atlantic	3.08 (2.41, 3.67)***	21.84
East North Central	3.49 (2.83, 4.15)***	32.80
West North Central	2.85 (2.09, 3.62)***	17.33
South Atlantic	3.32 (2.66, 3.98)***	27.71
East South Central	2.93 (2.19, 3.66)***	18.72
West South Central	2.27 (1.53, 3.02)***	9.72
Mountain	1.67 (0.90, 2.44)***	5.31
Pacific	1.73 (1.04, 2.41)***	5.62

* *p* < 0.05, ** *p* < 0.01, *** *p* < 0.001

**Table 8:** Linear regression model with Prais-Winsten estimator describing the relationship between a state’s total number of firearm laws and number of other states for which it is the source of 100 or more crime-related firearms in a given year, adjusting for state-level characteristics, 2010–2017

**Variable**	**b (95% CI)**
Intercept	−0.004 (−0.58, 0.57)
Year	0.08 (0.05, 0.10)***
Poverty rate	0.05 (−0.01, 0.11)
Density	−0.12 (−0.26, 0.06)
Area	0.70 (0.37, 1.02)***
Firearm ownership	−0.05 (−0.12, 0.01)***
Number of laws	−0.21 (−0.42, 0.00)*
Census division	
New England	ref
Mid-Atlantic	1.41 (0.52, 2.31)**
East North Central	0.75 (0.12, 1.39)*
West North Central	−0.02 (−0.71, 0.67)
South Atlantic	1.50 (0.89, 2.11)***
East South Central	1.02 (0.37, 1.68)**
West South Central	0.30 (−0.42, 1.02)
Mountain	−0.42 (−1.08, 0.25)
Pacific	0.66 (−0.11, 1.44)

* *p* < 0.05, ** *p* < 0.01, *** *p* < 0.001

**Table 9:** Linear regression model with Prais-Winsten estimator describing the relationship between a state’s total number of firearm laws and number of other states for which it is the destination of 100 or more crime-related firearms in a given year, adjusting for state-level characteristics, 2010–2017

**Variable**	**b (95% CI)**
Intercept	−1.16 (−2.17, − 0.15)*
Year	0.08 (0.04, 0.12)***
Poverty rate	0.06 (−0.03, 0.16)
Density	0.04 (−0.05, 0.13)
Area	0.47 (0.09, 0.85)*
Firearm ownership	−0.23 (− 0.55, 0.08)
Number of laws	0.51 (0.30, 0.71)***
Census division	
New England	ref
Mid-Atlantic	1.83 (0.89, 2.77)***
East North Central	1.99 (0.95, 3.03)***
West North Central	1.31 (0.16, 2.45)*
South Atlantic	2.30 (1.28, 3.32)***
East South Central	1.80 (0.69, 2.91)**
West South Central	1.64 (0.36, 2.92)*
Mountain	0.96 (−0.24, 2.16)
Pacific	1.39 (0.35, 2.43)**

* *p* < 0.05, ** *p* < 0.01, *** *p* < 0.001

## Abbreviations

ATF: Bureau of Alcohol, Tobacco, Firearms, and Explosives
