# Supplementary figures and images for: Firearm laws and the network of firearm movement among US states
Source: BMC Public Health. 2021 Oct 7;21:1803. doi: 10.1186/s12889-021-11772-y (PMC8499462; doi:10.1186/s12889-021-11772-y)

Appendix Figure 1.

(a)


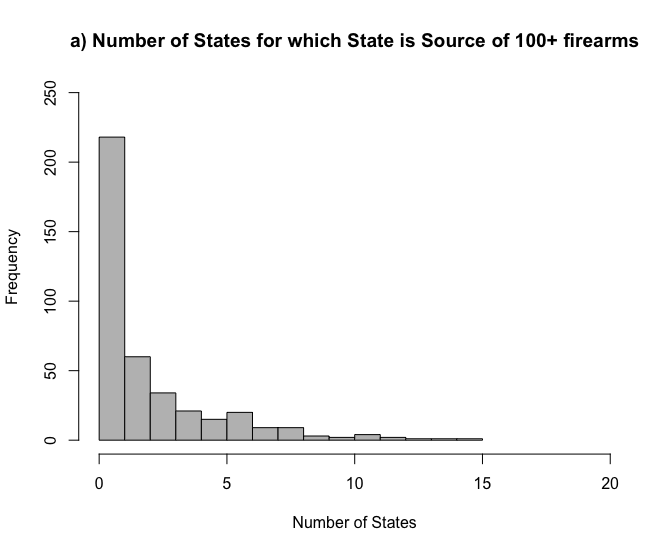


(b)


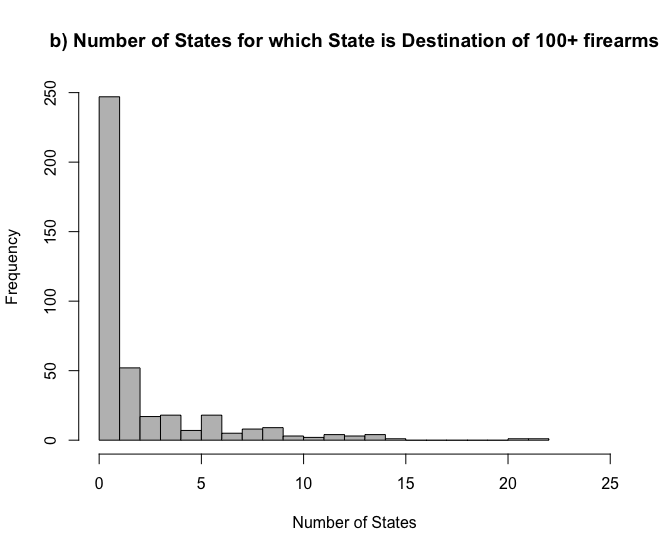


(c)


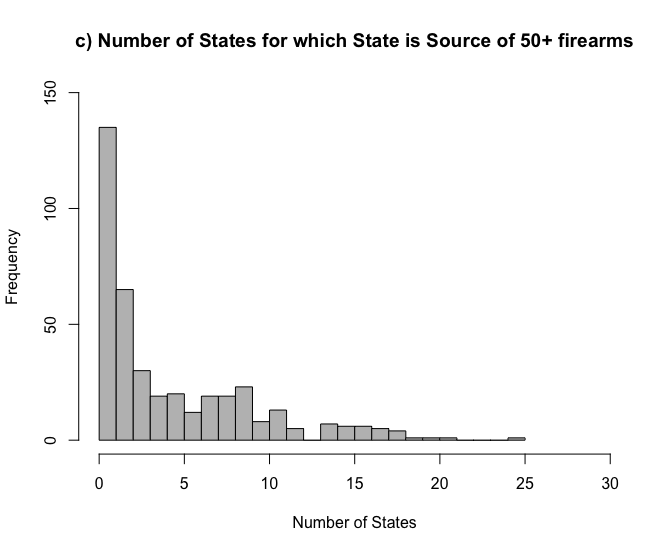


(d)

**
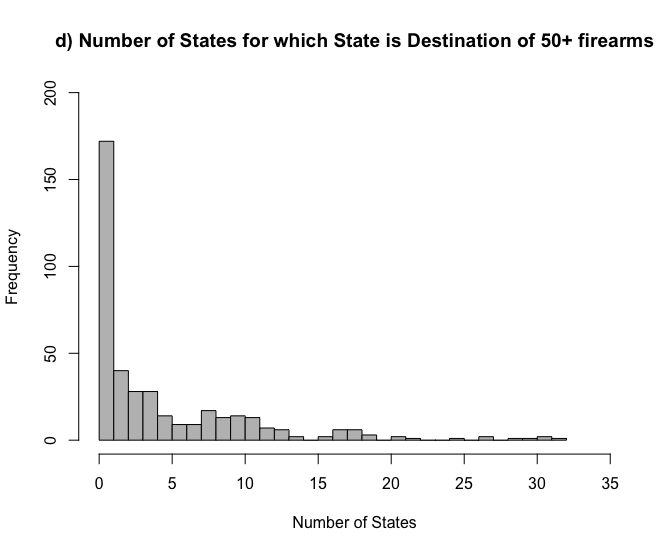
**

Supplement: Supplementary file 1 — Additional file 1: Figure S1. Histograms depicting the distribution of (A) the number of states for which states serve as sources of 100 or more firearms, (B) the number of states for which states serve as destinations of 100 or more firearms, (C) the number of states for which states serve as sources of 50 or more firearms, and (D) the number of states for which states serve as destinations of 50 or more firearms. [file 12889_2021_11772_MOESM1_ESM.docx]
